# *Agromyces arachidis* sp. nov. Isolated from a Peanut (*Arachis hypogaea*) Crop Field

**DOI:** 10.1155/2013/831308

**Published:** 2013-11-17

**Authors:** Chandandeep Kaur, Anil Kumar Pinnaka, Nitin Kumar Singh, Monu Bala, Shanmugam Mayilraj

**Affiliations:** Microbial Type Culture Collection and Gene Bank (MTCC), CSIR Institute of Microbial Technology (IMTECH), Sector 39-A, Chandigarh 160 036, India

## Abstract

A Gram-positive, yellowish bacterium strain AK-1^T^ was isolated from soil sample collected from peanut (*Arachis hypogaea*) crop field and studied by using a polyphasic approach. The organism had morphological and chemotaxonomic properties consistent with its classification in the genus *Agromyces*. Phylogenetic analysis of the 16S rRNA gene sequence showed that strain AK-1^T^ was closely related to *Agromyces aurantiacus* (98.6%) followed by *Agromyces soli* (98.3%), *Agromyces tropicus* (97.6%), *Agromyces ulmi* (97.3%), *Agromyces flavus* (97.2%), and *Agromyces italicus* (97.0%), whereas the sequence similarity values with respect to the other *Agromyces* species with validly published names were between 95.3 and 96.7** **%. However, the DNA-DNA hybridization values obtained between strain AK-1^T^ and other related strains were well below the threshold that is required for the proposal of a novel species. The DNA G + C content of the strain is 71.8 mol%. The above data in combination with the phenotypic distinctiveness of AK-1^T^ clearly indicate that the strain represents a novel species, for which the name *Agromyces arachidis* sp. nov. is proposed. The type strain is AK-1^T^ (=MTCC 10524^T^ = JCM 19251^T^).

## 1. Introduction

The genus *Agromyces* was first proposed by Gledhill and Casida Jr [1] and later on emended by Zgurskaya et al. [2]. At present, the genus *Agromyces *comprises 24 species with validly published names (http://www.bacterio.net/a/agromyces.html), and all these species have been isolated from different environmental sources: soils from fertile meadows, rhizosphere, and plants to rock art paintings [1–18]. In the present study, bacterial strain AK-1^T^, isolated from soil sample, is described and subjected to the polyphasic taxonomy. 16S rRNA gene sequence comparison revealed that the isolate is *Agromyces*-like organism. The aim of the present study is to determine the exact taxonomic position of the isolate.

## 2. Materials and Methods

Strain AK-1^T^ was isolated from a soil sample collected from peanut (*Arachis hypogaea*) crop field, Srikakulam, Andhra Pradesh, India (18°14′N latitude 83°58′E longitude), by the dilution-plate technique on tryptic soy agar medium (TSA; HiMedia) and maintained as glycerol stocks at −70* *°C. The reference strains *A. aurantiacus* (MTCC 11069^T^), *A. soli *(MTCC 11074^T^), *A. tropicus *(MTCC 11075^T^), *A. ulmi *(MTCC 10783^T^), *A. flavus* (MTCC 11103^T^), and *A. italicus *(MTCC 10784^T^) were obtained from the Microbial Type Culture Collection and Gene Bank (MTCC), Institute of Microbial Technology, Chandigarh, India.

Colony and cell morphologies were studied according to standard methods [19]. The Gram reaction was determined using the HiMedia Gram staining kit according to the manufacturer's instructions. Physiological tests like growth at different temperatures ranging from 10 to 55°C and NaCl concentrations (1–15%) were performed by growing the strain on TSA supplemented with different concentrations of NaCl. The pH range (5.0–12.0) and the optimum pH for growth were examined as described by Xu et al. [20] using TSB as basal medium. For anaerobiosis, the cultures were streaked on TSA plates and placed in an anaerobic jar (MART), which was evacuated and flushed with Anoxomat unit (MART) using anaerobic gas mixture consisting of nitrogen (85%), carbon dioxide (10%), and hydrogen (5%). Plates were incubated at 30°C for 5 days. Catalase activity, citrate utilization (using Simmons' citrate agar), and urea hydrolysis were determined as described by Cowan and Steel [21]. The hydrolysis of casein, gelatin, Tween 80, tyrosine, starch and indole, methyl red test, Voges-Proskauer test, and oxidase activity were assessed as described by Smibert and Krieg [22]. Nitrate reduction was tested as described by Lányi [23]. VITEK 2-GP cards were used as per the instructions of the manufacturer (bioMérieux). Acid production from various sugars was tested on minimal medium by using the method described by Smith et al. [24].

For cellular fatty acid analysis, the strains were grown on TSA medium at 30* *°C for 36 h; fatty acids were saponified, methylated, and extracted using the standard protocol of MIDI (Sherlock Microbial Identification System, version 4.0). The fatty acids were analysed by GC (Hewlett Packard 6890) and identified by using the TSBA50 database of the Microbial Identification System as described by Sasser [25] and Pandey et al. [26]. Freeze-dried cells for other chemotaxonomic analyses were prepared following growth of the strains in tryptic soy broth for 4days at 30°C. The peptidoglycan structure was determined by using a hydrolysate of purified cell walls, according to Schleifer [27]. The diagnostic amino acids were separated by single dimensional ascending TLC as described by Schleifer and Kandler [28], with the modification that TLC on cellulose sheets (Merck 5577) was used instead of paper chromatography. Polar lipids and menaquinones were extracted and analysed by using the methods described by Minnikin et al. [29] and Kroppenstedt [30].

Genomic DNA extraction, amplification, and sequencing were performed as described previously by Mayilraj et al. [31]. The complete sequence of the 16S rRNA gene was aligned with those of representative related taxa using the EzTaxon server (http://www.eztaxon.org/) [32]. The 16S rRNA gene sequence of AK-1^T^ and the representative of closely related species were retrieved from the EzTaxon server and aligned using MEGA version 5.0 [32]. Phylogenetic trees were constructed using the neighbour-joining as well as maximum parsimony algorithms and maximum likelihood algorithms. Bootstrap analysis was performed to assess the confidence limits of the branching. DNA-DNA hybridization was performed by the membrane filter method [33]. The G + C content of the genomic DNA was determined spectrophotometrically (Lambda 35; Perkin Elmer) using the thermal denaturation method [34].

## 3. Results and Discussion

**Figure 1:**
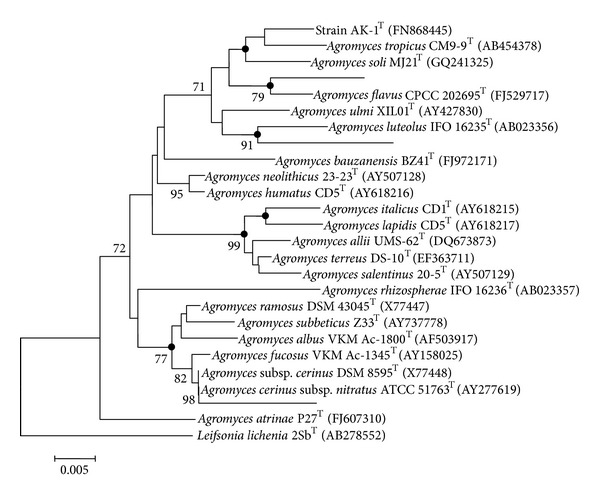
Phylogenetic neighbour-joining tree based on 16S rRNA gene sequences (1442 bases) showing the relationship between *Agromyces arachidis *AK-1^T^ and related members of the genus *Agromyces. Leifsonia lichenia* 2Sb^T^ (AB278552) was used as an outgroup. Bootstrap values (expressed as percentages of 1000 replications) greater than 70% are given at nodes. Filled circles indicate that corresponding nodes were also recovered in the tree generated with maximum parsimony and maximum likelihood algorithms. Bar, 0.005% sequence variation. GenBank accession numbers are given in parentheses.

**Figure 2:**
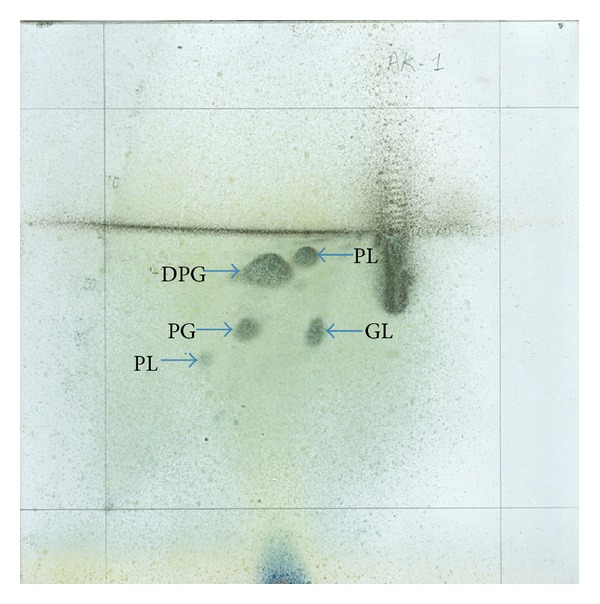
Two-dimensional thin layer chromatograms of the total lipids of strain AK-1^T^, detected with molybdophosphoric acid (5% w/v) in absolute ethanol. Phosphatidylglycerol (PG), diphosphatidylglycerol (DPG), unknown phospholipid (PL), and unknown glycolipids (GL).

Detailed phenotypic properties that differentiate strain AK-1^T^ from closely related species of the genus *Agromyces *are summarized in Table 1. Most of the chemotaxonomic properties, including the fatty acid composition, were typical of members of the genus *Agromyces*. The major menaquinone detected for the strain AK-1^T^ is MK-12 (54.13%), while MK-11 (14.08%) and MK-13 (31.77%) are the other minor components; major fatty acids are anteiso-C_15:0_, anteiso-C_17:0_, iso-C_15:0_, and iso-C_16:0_ (Table 2); cell wall diagnostic amino acid is 2,4-diaminobutyric acid. Major lipids are diphosphatidylglycerol (DPG), phosphatidylglycerol (PG), two unknown phospholipids, and one unknown glycolipid (Figure 2). The almost complete 16S rRNA gene sequence of strain AK-1^T^ (1442 bases) was determined. Phylogenetic analysis of the 16S rRNA gene sequence showed that strain AK-1^T^ was closely related to *A. aurantiacus* (98.6%) followed by *A. soli* (98.3%), *A. tropicus* (97.6%), *A. ulmi* (97.3%), *A. flavus* (97.2%), and *A. italicus* (97.0%). The similarities with respect to the type strains of the remaining species of the genus were significantly lower (95.3–96.7* *%). The 16S rRNA gene sequence-based phylogenetic analysis revealed that strain AK-1^T^ forms a separate branch within the lineage that includes *A. aurantiacus*, *A. soli*, *A. tropicus*, *A. ulmi*, and *A. flavus* (Figure1); this was also evident in the phylogenetic tree constructed using maximum parsimony and maximum likelihood algorithms (shown as closed circles at the nodes in Figure 1) where the strain was recovered as a separate clade. The DNA-DNA hybridization values for strain AK-1^T^ with the closely related species were less than 56.2%, which is well below the 70* *% threshold value recommended for the delineation of bacterial species [35]. The levels of DNA-DNA relatedness between strain AK-1^T^ and other *Agromyces *species were not determined, since it has been shown that organisms with more than 3* *% 16S rRNA gene sequence dissimilar belong to different genomic species [36]. On the basis of the polyphasic data presented previously, strain AK-1^T^ should be placed in the genus *Agromyces* within a novel species, for which we propose the name *Agromyces arachidis*sp. nov. 

### 3.1. Description of Agromyces arachidis sp. nov.

*Agromyces arachidis* sp. nov. (a.ra′ chi. dis. N. L. n. Arachis-idis, a botanical generic name; N. L. gen. n. *arachidis*, of Arachis, isolated from a peanut (*Arachis hypogaea*) crop field).

The cells are Gram-positive, strictly aerobic, nonspore forming, and occurring in straight or curved rods. Colonies are yellowish, opaque, convex, entire and 1-2 mm in diameter on tryptic soy agar medium, and capable of growing from 25°C to 37°C, with optimum for growth at 30°C and a pH range from 6.0 to 10.0; they can tolerate up to 1.0% NaCl. Strain AK-1^T^ shows positive reaction for hydrolysis of starch and negative for casein hydrolysis, urease production, MR-VP reaction, hydrogen sulphide production, and nitrate reduction. Acid is produced from arabinose, xylose, inulin, and lactose; it is negative for salicin, mannitol, melibiose, galactose, sucrose, rhamnose, trehalose, mannose, maltose, and raffinose. Other detailed characteristics features are mentioned in Table 1. Major polar lipids are phosphatidylglycerol (PG) and diphosphatidylglycerol (DPG), two unknown phospholipids (PL), and one unknown glycolipid (GL). The major menaquinone detected for the strain AK-1^T^ is MK-12 (54.1%), while MK-13 (31.7%) and MK-11 (14.0%) are the other components. The predominant fatty acids are anteiso-C_15:0_, anteiso-C_17:0_, iso-C_15:0_, and iso-C_16:0_. The diagnostic diamino acid in cell wall hydrolyzate is 2,4-diaminobutyric acid. The DNA G + C content of the strain is 71.8 mol%. The type strain, AK-1^T^ (=MTCC 10524^T^ = JCM 19251^T^), was isolated from a soil sample collected from peanut (*Arachis hypogaea*) crop field, Srikakulam, Andhra Pradesh, India.

## Figures and Tables

**Table 1:** Differential characteristics that differentiate strain AK-1^T^ along with the closest species AK-1^T ^(MTCC 10524^T^), *A. aurantiacus* (MTCC 11069^T^), *A. soli *(MTCC 11074^T^), *A. tropicus *(MTCC 11075^T^), *A. ulmi *(MTCC 10783^T^), *A. flavus* (MTCC 11103^T^), and *A. italicus *(MTCC 10784^T^).

Characteristics	AK-1^T^ (MTCC 10524^T^)	*A. aurantiacus* (MTCC 11069^T^)	*A. soli *(MTCC 11074^T^)	*A. tropicus *(MTCC 11075^T^)	*A. ulmi *(MTCC 10783^T^)	*A. flavus* (MTCC 11103^T^)	*A. italicus *(MTCC 10784^T^)
Growth at							
37°C	+	+	+	+	−	+	+
42°C	−	−	+	−	−	−	−
2% NaCl	−	−	+	+	−	−	+
5% NaCl	−	−	+	−	−	−	+
pH 5.0	+	−	−	+	+	+	+
pH 10.0	−	+	+	+	−	−	+
pH 12.0	−	−	+	+	−	−	+
Starch hydrolysis	+	+	−	+	−	+	−
Casein hydrolysis	−	−	−	−	−	+	−
Urease	−	+	−	−	−	−	−
Catalase	−	+	+	+	−	+	−
Acid production from carbohydrates							
Salicin	−	+	+	+	+	−	+
Mannitol	−	−	−	+	+	+	−
Melibiose	−	−	−	+	−	−	−
Galactose	−	−	+	+	+	−	+
Arabinose	+	−	−	+	−	+	−
Cellobiose	+	−	+	+	+	−	+
Sucrose	−	+	+	+	+	−	+
Xylose	+	−	−	+	+	+	−
Inositol	−	−	−	−	−	+	−
Rhamnose	−	−	+	+	+	−	+
Lactose	+	+	−	+	−	−	−
Trehalose	+	+	+	+	+	−	−
Mannose	−	+	+	+	+	+	+
Maltose	−	−	+	+	+	−	+
Raffinose	−	+	+	+	+	−	+
Sensitivity to antibiotics (*µ*g/disc)							
Nitrofurantoin (300)	S	R	R	S	S	S	S
Norfloxacin (10)	R	S	R	R	R	S	R
Polymyxin B (300)	S	S	R	S	S	S	R
Kanamycin (30)	S	S	R	S	S	S	R
Colistin (10)	R	R	R	S	R	S	S
Methicillin (5)	R	S	R	S	R	S	S
Oxacillin (5)	R	S	R	S	R	S	S
Gentamycin (10)	S	R	S	S	S	S	S
Trimethoprim (5)	S	S	R	S	S	S	S
Oxytetracycline (30)	S	S	R	S	S	S	R
Cefoxitin (30)	S	S	R	S	R	S	S
Biochemical tests using VITEK 2GP card							
Arginine dihydrolase 1	−	−	−	−	−	−	+
Leucine arylamidase	+	+	+	+	+	+	+
*α*-Glucosidase	+	+	+	+	−	−	+
L-Proline arylamidase	+	+	+	+	−	+	+
*α*-Galactosidase	−	+	−	+	−	−	−
Alanine arylamidase	+	+	+	+	−	+	+
Tyrosine arylamidase	−	+	+	−	−	+	+
L-Lactate alkalinization	−	−	−	−	−	−	+
Salicin	−	−	−	−	−	−	+
Quinone type	MK12 11, 13	MK12, 13	MH12	MK12	M12, 11, 10	MK12	MK12, 13
Total lipid pattern	DPG, PG	DPG, PG	DPG, PG	DPG, PG	DPG, PG	DPG, PG	DPG, PG
DNA G + C mol%	71.8	72.8	73.4	72.7	72.0	70.9	70.8

All the strains were positive at pH 8.0 and 9.0, at temperatures 25°C and 30°C, and acid production from fructose; negative at 12°C, 10%, 15% NaCl, dulcitol, inositol, sorbitol, adonitol, citrate, methyl-red, Voges-Proskauer, indole, nitrate, and gelatin liquefaction. All the strains are negative for the following biochemical tests using VITEK 2-GP card: D-Amygdalin, phosphatidylinositol phospholipase C, D-xylose, *β*-galactosidase, Ala-Phe-Pro-arylamidase, cyclodextrin, L-aspartate arylamidase, *β*-galactopyranosidase, *α*-mannosidase, phosphatase, *β*-glucuronidase, L-pyrrolldonyl arylamidase, D-sorbitol, urease, polymyxin B resistance, D-galactose, D-ribose, lactose, N-acetyl-D-glucosamine, D-maltose, bacitracin resistance, novobiocin resistance, growth in 6.5% NaCl, D-mannitol, D-mannose, methyl-*β*-D-glucopyranoside, pullulan, D-raffinose, O/129 resistance (comp. vibrio.), sucrose, D-trehalose, arginine dihydrolase 2 and optochin resistance. All the strains were sensitive to triple sulphas, kanamycin, sulfonamide, novobiocin, ampicillin, and rifampicin. S: sensitive; R: resistance.

**Table 2:** Percentage of total cellular fatty acids from strains AK-1^T ^(MTCC 10524^T^), * A. aurantiacus* (MTCC 11069^T^), *A. soli *(MTCC 11074^T^), *A. tropicus *(MTCC 11075^T^), *A. ulmi *(MTCC 10783^T^), *A. flavus* (MTCC 11103^T^), and *A. italicus *(MTCC 10784^T^).

Type of fatty acids	AK-1^T^ (MTCC 10524^T^)	*A. aurantiacus* (MTCC 11069^T^)	*A. soli* (MTCC 11074^T^)	*A. tropicus *(MTCC 11075^T^)	*A. ulmi* (MTCC 10783^T^)	*A. flavus* (MTCC 11103^T^)	*A. italicus* (MTCC 10784^T^)
iso-C14:0	0.8	0.7	0.6	3.0	2.5	1.4	0.6
iso-C15:0	9.4	7.7	6.1	3.6	15.5	2.8	6.3
anteiso C15:0	47.7	32.7	39.5	31.9	58.3	41.5	40.2
iso-C16:0	11.3	11.4	18.1	32.7	1.8	23.6	18.6
C16:0	0.9	2.8	0.6	0.5	2.5	0.5	0.7
iso-C17:0	3.3	3.0	1.8	3.3	3.0	0.7	1.7
anteiso C 17:0	21.86	31.6	31.2	20.6	1.6	23.7	29.2
C18:0	tr	0.6	tr	tr	1.5	tr	tr
C18:3 *ω*6c	ND	0.7	ND	ND	1.3	0.7	tr
iso-C19:0	ND	tr	tr	ND	tr	1.0	ND

Data from the present study. Fatty acids amounting to <0.5% of the total fatty acids in all strains are not shown or shown as tr: traces. ND: not detected.
